# The Design of GNSS/IMU Loosely-Coupled Integration Filter for Wearable EPTS of Football Players

**DOI:** 10.3390/s23041749

**Published:** 2023-02-04

**Authors:** Mingu Kim, Chulwoo Park, Jinsung Yoon

**Affiliations:** 1 Division of Mechanical and Electronics Engineering, Hansung University, Seoul 02876, Republic of Korea; 2 Fitogether Inc., Seoul 04378, Republic of Korea

**Keywords:** EPTS, GNSS, IMU, integration filter, extended kalman filter, sport science

## Abstract

This study presents the filter design of GNSS/IMU integration for wearable EPTS (Electronic Performance and Tracking System) of football players. EPTS has been widely used in sports fields recently, and GNSS (Global Navigation Satellite System) and IMU (Inertial Measurement Unit) in wearable EPTS have been used to measure and provide players’ athletic performance data. A sensor fusion technique can be used to provide high-quality analysis data of athletic performance. For this reason, the integration filter of GNSS data and IMU data is designed in this study. The loosely-coupled strategy is considered to integrate GNSS and IMU data considering the specification of the wearable EPTS product. Quaternion is used to estimate a player’s attitude to avoid the gimbal lock singularity in this study. Experiment results validate the performance of the proposed GNSS/IMU loosely-coupled integration filter for wearable EPTS of football players.

## 1. Introduction

Collecting athletic information with professional sports teams is becoming increasingly critical. Athletic data obtained during training sessions or match games have been used to analyze and monitor players’ performance. The analysis results of athletic data can be used to provide a training guideline to improve players’ performance [1,2,3,4]. Moreover, analyzing athletic data can help to reduce injury risks [5,6].

Video- and computer-aided analysis is a method to analyze the athletic performance of numerous professional and international sports teams. The development of video and computer technology enhances the quality of athletic data and match information, and sports broadcasting programs can provide visualized data for TV audiences [7,8,9,10,11,12].

Motion analysis becomes important for improving athlete performance and reducing athletes’ injury risk. IMU (Inertial Measurement Unit) sensors which consist of three-axis accelerometers, three-axis gyroscopes, and three-axis magnetometers have been used to estimate and provide the attitude, position, and velocity of athletes [13,14,15,16,17,18,19,20,21,22,23]. The head or foot injuries of sports players can be monitored by analyzing G-impacts and reaction forces using the measured acceleration data from IMU sensors [13,14,15]. The different IMU sensor positions can be possible to provide various physical load estimates of athletes and analysis the motion of athletes, i.e., football players movement intensity information [16], runners’ stride length and stride velocity, analysis at ground contacts [17], postural demands of professional soccer players [18], velocity measurements for team sports [19], and the analysis of foot swing at football kicks [20]. Deep learning techniques using IMU sensor information were also used to classify football activities [21,22,23].

Navigation data (i.e., position and velocity) can be obtained from IMU sensors. However, IMU sensors providing reliable and accurate navigation data are costly, and navigation performance using only IMU sensors degrades gradually because the integration error becomes significant. Therefore, high-cost IMU sensors are not suitable for the application of wearable equipment in sports science. Accordingly, GPS (Global Positioning Systems) is a suitable option for tracking the position and velocities of athletes in outdoor sports. GPS was first developed for military purposes by the US government. GPS has been widely used in various civil and commercial fields after eliminating the SA (Selective Availability) that degrades public GPS signals. In addition to GPS, several satellite navigation systems, such as the European Galileo, Russian GLONASS (GLObal NAvigaion Satellite System), Chinese Beidou, and Japanese QZSS (Quasi-Zenith Satellite System), have been developed and widely used to navigate vehicles. These GNSS (Global Navigation Satellite System) signals can be used to track athlete data, particularly movements and velocities during outdoor training and matches [24,25,26,27,28].

GNSS provides position and velocity data with a bounded error; the GNSS data rate is comparatively lower than the IMU data rate. The interpolation of GNSS data can be an option to obtain smooth trajectories. However, sports players can move quickly during match games or training sessions. In the situation, i.e., the agile behavior of sports players, the simple interpolation of GNSS data cannot provide accurate performance. Furthermore, GNSS data cannot be obtained when the number of visible satellites decreases beyond a specific number. Accordingly, the GNSS/IMU integration can be a more suitable solution for wearable EPTS of football player than the simple interpolation of GNSS data.

The integration of GNSS and IMU data has been widely studied, especially in vehicle navigation [29,30,31,32,33,34,35,36,37,38,39,40,41,42,43,44,45]. GNSS/IMU integration architectures can be categorized into loosely-coupled, tightly-coupled, and deep [46]. The deep integration of GNSS/IMU can be achieved under the condition that the baseband signal processing of GNSS is possible. Therefore, loosely and the tightly-coupled integrations of GNSS/IMU have been typically used for commercial GNSS and IMU modules. The principle of the loosely-coupled integration of GNSS/IMU blends the position and velocities of GNSS data and the navigation data provided by inertial sensors. In contrast, the tightly-coupled integration of GNSS/IMU needs GNSS raw measurements (i.e., pseudorange and Doppler observables). Although the tightly-coupled integration of GNSS/IMU has the advantage that navigation data with a poor GNSS signal is available, the tightly-coupled integration technique is more complex than the loosely-coupled integration and requires more effort because of the use of GNSS raw measurements. Therefore, the loosely-coupled integration of GNSS/IMU is suitable for low-cost GNSS and IMU modules, such as wearable device applications.

Many studies on sensor technology have been performed in sports science [13,14,15,16,17,18,20,21,22,23,24,25,26,27,28,47], but few on GNSS/IMU integration have been performed [21,47]. In this study, we investigate the loosely-coupled integration of GNSS/IMU for a wearable EPTS with football players because most commercial wearable EPTS systems do not provide GNSS raw measurement data (e.g., pseudorange). GNSS data can be obtained in outdoor environments, and football is one of the representative outdoor sports. Therefore, this study focuses on applying a wearable EPTS for football players. A Kalman filter is a representative sensor fusion technique for linear systems. However, the kinematics considered in integrating GNSS/IMU are nonlinear. Therefore, EKFs (Extended Kalman Filters) are used to integrate the GNSS and IMU data to provide the performance data of football players. Quaternion is used to estimate an athlete’s attitude to avoid gimbal lock singularity. We first use a Kalman filter to obtain the athlete’s attitude from accelerometer and gyroscope data. we then use an EKF to integrate the navigation data of GNSS and the position, velocity, and attitude data of IMU. We perform experiments to validate the performance of the proposed GNSS/IMU loosely-coupled integration filter for a wearable EPTS for football players. We compare the performance of the proposed integration filter and the performance of the commercial high-precision GNSS/IMU AHRS (Attitude Heading Reference System). We also analyze the performance of the designed integration filter compared with data obtained from a Vicon motion capture system—popular, highly accurate equipment generally used to analyze the motion of the athletes using markers.

The remainder of this paper is organized as follows. Section 2 presents the architecture of the GNSS/IMU loosely-coupled integration filter for a wearable EPTS for football players. Section 3 presents the experimental results to validate the performance of the proposed GNSS/IMU loosely-coupled integration filter. Concluding remarks are presented in Section 4.

## 2. GNSS/IMU Loosely-Coupled Integration Filter Design

**Figure 1:**
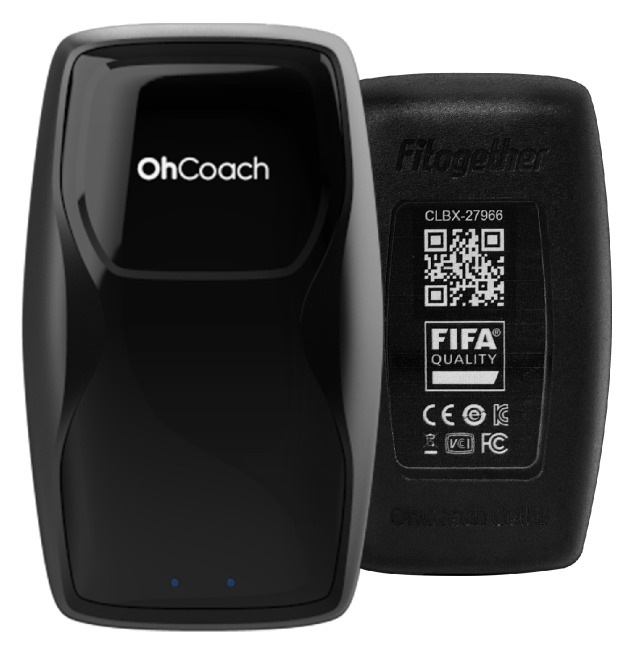
A wearable EPTS of football players.

This section presents the proposed GNSS (Global Navigation Satellite Systems)/IMU (Inertial Measurement Unit) loosely-coupled integration filter for wearable EPTS (Electronic Performance and Tracking Systems) of football players. In this study, we consider a commercial wearable EPTS for football players. The considered wearable EPTS provides position and velocity data of GNSS but do not provide GNSS raw measurement data (e.g., pseudorange). Therefore, the loosely-coupled integration scheme is suitable for a wearable EPTS for football players. The EPTS is equipped with an MPU-9250 which is an IMU and a U-blox NEO-M8 GNSS receiver. The GNSS data rate is 10 Hz and the IMU data rate is 100 Hz. The horizontal position and velocity accuracies of the GNSS are 2.5 m and 0.05 m · s^−1^. Figure 1 shows the wearable EPTS used in this study.

### 2.1. The Preliminaries of Attitude Estimation

Euler angles are used to describe the rotation of the rigid body, i.e., roll angle (ϕ), pitch angle (θ), and yaw angle (ψ). However, the singularity can occur, especially, when the pitch angle is $\theta =\pm 90^{{}^{\circ}}$. To overcome the singularity issue, quaternion was introduced. The quaternion is defined as

(1) $$\vec{q}=q_{0}+q_{1}\vec{i}+q_{2}\vec{j}+q_{3}\vec{k}$$

The relation between quaternion and Euler angles and the quaternion dynamics can be written as

(2) $$q_{0}=\pm \left(\cos\frac{\phi }{2}\cos\frac{\theta }{2}\cos\frac{\psi }{2}+\sin\frac{\phi }{2}\sin\frac{\theta }{2}\sin\frac{\psi }{2}\right)$$

(3) $$q_{1}=\pm \left(\sin\frac{\phi }{2}\cos\frac{\theta }{2}\cos\frac{\psi }{2}-\cos\frac{\phi }{2}\sin\frac{\theta }{2}\sin\frac{\psi }{2}\right)$$

(4) $$q_{2}=\pm \left(\cos\frac{\phi }{2}\sin\frac{\theta }{2}\cos\frac{\psi }{2}+\sin\frac{\phi }{2}\cos\frac{\theta }{2}\sin\frac{\psi }{2}\right)$$

(5) $$q_{3}=\pm \left(\cos\frac{\phi }{2}\cos\frac{\theta }{2}\sin\frac{\psi }{2}-\sin\frac{\phi }{2}\sin\frac{\theta }{2}\cos\frac{\psi }{2}\right)$$

(6) $$\left[\begin{array}{c} {\dot{q}}_{0}\\ {\dot{q}}_{1}\\ {\dot{q}}_{2}\\ {\dot{q}}_{3} \end{array}\right]=\frac{1}{2}\left[\begin{array}{ccc} -q_{1} & -q_{2} & -q_{3}\\ q_{0} & -q_{3} & q_{2}\\ q_{3} & q_{0} & -q_{1}\\ -q_{2} & q_{1} & q_{0} \end{array}\right]\left[\begin{array}{c} p\\ q\\ r \end{array}\right]$$

where the rotation angular velocity of the rigid body is $\vec{\omega }=p{\vec{i}}_{B}+q{\vec{j}}_{B}+r{\vec{k}}_{B}$ and ${\vec{i}}_{B}$, ${\vec{j}}_{B}$, and ${\vec{k}}_{B}$ are the unit vectors in the body frame.

The attitude of the rigid body can be obtained from an accelerometer as follows,

(7) $$\phi =\tan^{-1}\frac{a_{y}}{a_{z}}$$

(8) $$\theta =\tan^{-1}\frac{a_{x}}{\sqrt{a_{y}^{2}+a_{z}^{2}}}$$

where $a_{x}$, $a_{y}$, and $a_{z}$ are the accelerations measured from an accelerometer. Note that yaw angle cannot be obtained from an accelerometer.

The attitude of the rigid body can be also obtained by integrating the angular rate calculated using gyroscope data. Yaw angle information can be obtained from gyroscope data.

(9) $$\left[\begin{array}{c} \dot{\phi }\\ \dot{\theta }\\ \dot{\psi } \end{array}\right]=\left[\begin{array}{ccc} 1 & \sin\phi \tan\theta & \cos\phi \tan\theta \\ 0 & \cos\phi & -\sin\phi \\ 0 & \sin\phi \sec\theta & \cos\phi \sec\theta \end{array}\right]\left[\begin{array}{c} p\\ q\\ r \end{array}\right]$$

### 2.2. The GNSS/IMU Loosely-Coupled Integration

**Figure 2:**
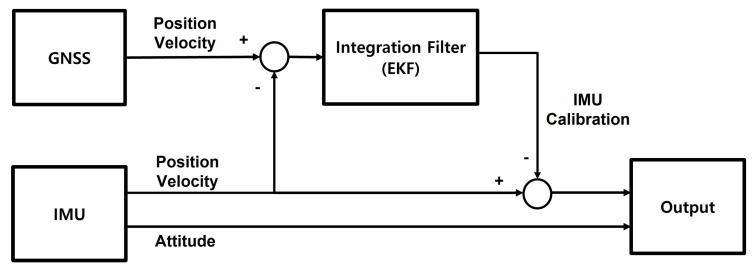
The block diagram of the GNSS/IMU loosely-coupled integration filter.

The block diagram of GNSS/IMU loosely-coupled integration filter designed in the study is shown as Figure 2. The navigation data, i.e., position and velocity, obtained from GNSS signal are blended with the navigation data obtained from IMU measurement using an EKF (Extended Kalman Filter). The attitude data is obtained from an IMU sensor.

The state vector of the designed GNSS/IMU loosely-coupled integration filter is defined as

(10) $$x={\left[x,y,z,V_{x},V_{y},V_{z},q_{0},q_{1},q_{2},q_{3}\right]}^{T}$$

where *x*, *y*, *z*, $V_{x}$, $V_{y}$, and $V_{z}$ are the positions and velocities of an athlete in the local frame, respectively, and $q_{0}$, $q_{1}$, $q_{2}$, and $q_{3}$ are the quaternions for representing the attitude of an athlete. The input vector of the loosely-coupled integration filter is defined as

(11) $$u={\left[a_{x},a_{y},a_{z},p,q,r\right]}^{T}$$

where $a_{x}$, $a_{y}$, $a_{z}$, *p*, *q*, and *r* are the acceleration and angular rates of an athlete in the body frame, respectively.

The attitude update equation using quaternion can be represented as Equation (6), and the velocity update equation is written as Equation (12) using the DCM (Direction Cosine Matrix) and quaternion.

(12) $$\left[\begin{array}{c} {\dot{V}}_{x}\\ {\dot{V}}_{y}\\ {\dot{V}}_{z}+g \end{array}\right]=\left[\begin{array}{ccc} q_{0}^{2}+q_{1}^{2}-q_{2}^{2}-q_{3}^{2} & 2\left(q_{1}q_{2}-q_{0}q_{3}\right) & 2\left(q_{1}q_{3}+q_{0}q_{2}\right)\\ 2\left(q_{1}q_{2}+q_{0}q_{3}\right) & q_{0}^{2}-q_{1}^{2}+q_{2}^{2}-q_{3}^{2} & 2\left(q_{2}q_{3}+q_{0}q_{1}\right)\\ 2\left(q_{1}q_{3}-q_{0}q_{2}\right) & 2\left(q_{2}q_{3}+q_{0}q_{1}\right) & q_{0}^{2}-q_{1}^{2}-q_{2}^{2}+q_{3}^{2} \end{array}\right]\left[\begin{array}{c} a_{x}\\ a_{y}\\ a_{z} \end{array}\right]$$

where *g* is the gravitational acceleration.

The state update nonlinear equation, $\dot{x}=f\left(x,u,w\right)$, can be rewritten as Equation (13) using the gravitational acceleration and error covariance matrix.

(13) $$\begin{array}{c} \dot{x}=\left[\begin{array}{c} V_{x}\\ V_{y}\\ V_{z}\\ a_{x}\left(q_{0}^{2}+q_{1}^{2}-q_{2}^{2}-q_{3}^{2}\right)+2a_{y}\left(q_{1}q_{2}-q_{0}q_{3}\right)+2a_{z}\left(q_{1}q_{3}+q_{0}q_{2}\right)\\ 2a_{x}\left(q_{1}q_{2}+q_{0}q_{3}\right)+a_{y}\left(q_{0}^{2}-q_{1}^{2}+q_{2}^{2}-q_{3}^{2}\right)+2a_{z}\left(q_{2}q_{3}-q_{0}q_{1}\right)\\ 2a_{x}\left(q_{1}q_{3}-q_{0}q_{2}\right)+2a_{y}\left(q_{2}q_{3}+q_{0}q_{1}\right)+a_{z}\left(q_{0}^{2}-q_{1}^{2}-q_{2}^{2}+q_{3}^{2}\right)-g\\ -\frac{1}{2}pq_{1}-\frac{1}{2}qq_{2}-\frac{1}{2}rq_{3}\\ \frac{1}{2}pq_{0}-\frac{1}{2}qq_{3}+\frac{1}{2}rq_{2}\\ \frac{1}{2}pq_{3}+\frac{1}{2}qq_{0}-\frac{1}{2}rq_{1}\\ -\frac{1}{2}pq_{2}+\frac{1}{2}qq_{1}+\frac{1}{2}rq_{0} \end{array}\right]+w \end{array}$$

where $w∼N\left(0,Q\right)$, and Q is the covariance matrix of an IMU.

The EKF for GNSS/IMU loosely-coupled integration can be written as:

Prediction equations

(14) $${\hat{x}}_{k}^{-}=f\left({\hat{x}}_{k-1},u_{k-1},0\right)$$

(15) $$P_{k}^{-}=A_{k}P_{k-1}A_{k}^{T}+Q$$

Update equations

(16) $$K_{k}=P_{k}^{-}H_{k}^{T}{\left(H_{k}P_{k}^{-}H_{k}^{T}+R\right)}^{-1}$$

(17) $${\hat{x}}_{k}={\hat{x}}_{k}^{-}+K_{k}\left(z_{k}-h\left({\hat{x}}_{k}^{-},0\right)\right)$$

(18) $$P_{k}=\left(1-K_{k}H_{k}\right)P_{k}^{-}$$

Measurement equations

(19) z=Hx+v

(20) $$H=\left[\begin{array}{cccccccccc} 1 & 0 & 0 & 0 & 0 & 0 & 0 & 0 & 0 & 0\\ 0 & 1 & 0 & 0 & 0 & 0 & 0 & 0 & 0 & 0\\ 0 & 0 & 1 & 0 & 0 & 0 & 0 & 0 & 0 & 0\\ 0 & 0 & 0 & 1 & 0 & 0 & 0 & 0 & 0 & 0\\ 0 & 0 & 0 & 0 & 1 & 0 & 0 & 0 & 0 & 0\\ 0 & 0 & 0 & 0 & 0 & 1 & 0 & 0 & 0 & 0 \end{array}\right]$$

where *k* denotes the *k*-th time step, ${\hat{x}}_{k}$ is the estimate of the state vector at the *k*-th time step, ${\hat{x}}_{k}^{-}$ is the priori estimate of the state vector at the *k*-th time step, P is the error covariance matrix, K is Kalman gains, z is the position and velocity measured by GNSS, $h\left({\hat{x}}_{k-1}^{-},0\right)$ is the nonlinear observation equation, H is the observation matrix, $v∼N\left(0,R\right)$, and R denotes the covariance matrix of GNSS, respectively.

The matrix, $A_{k}$, is written as

(21) $$A_{k}=\left[\begin{array}{cccccccccc} 1 & 0 & 0 & T_{s} & 0 & 0 & 0 & 0 & 0 & 0\\ 0 & 1 & 0 & 0 & T_{s} & 0 & 0 & 0 & 0 & 0\\ 0 & 0 & 1 & 0 & 0 & T_{s} & 0 & 0 & 0 & 0\\ 0 & 0 & 0 & 1 & 0 & 0 & A_{47} & A_{48} & A_{49} & A_{4a}\\ 0 & 0 & 0 & 0 & 1 & 0 & A_{57} & A_{58} & A_{59} & A_{5a}\\ 0 & 0 & 0 & 0 & 0 & 1 & A_{67} & A_{68} & A_{69} & A_{6a}\\ 0 & 0 & 0 & 0 & 0 & 0 & 1 & A_{78} & A_{79} & A_{7a}\\ 0 & 0 & 0 & 0 & 0 & 0 & A_{87} & 1 & A_{89} & A_{8a}\\ 0 & 0 & 0 & 0 & 0 & 0 & A_{97} & A_{98} & 1 & A_{9a}\\ 0 & 0 & 0 & 0 & 0 & 0 & A_{a7} & A_{a8} & A_{a9} & 1 \end{array}\right]$$

where

(22) $$A_{47}=2T_{s}\left({\hat{a}}_{x_{k-1}}{\hat{q}}_{0_{k-1}}+{\hat{a}}_{y_{k-1}}{\hat{q}}_{3_{k-1}}-{\hat{a}}_{z_{k-1}}{\hat{q}}_{2_{k-1}}\right)$$

(23) $$A_{48}=2T_{s}\left({\hat{a}}_{x_{k-1}}{\hat{q}}_{1_{k-1}}+{\hat{a}}_{y_{k-1}}{\hat{q}}_{2_{k-1}}+{\hat{a}}_{z_{k-1}}{\hat{q}}_{3_{k-1}}\right)\;$$

(24) $$A_{49}=2T_{s}\left(-{\hat{a}}_{x_{k-1}}{\hat{q}}_{2_{k-1}}+{\hat{a}}_{y_{k-1}}{\hat{q}}_{1_{k-1}}-{\hat{a}}_{z_{k-1}}{\hat{q}}_{0_{k-1}}\right)$$

(25) $$A_{4a}=2T_{s}\left(-{\hat{a}}_{x_{k-1}}{\hat{q}}_{3_{k-1}}+{\hat{a}}_{y_{k-1}}{\hat{q}}_{0_{k-1}}+{\hat{a}}_{z_{k-1}}{\hat{q}}_{1_{k-1}}\right)$$

(26) $$A_{57}=2T_{s}\left(-{\hat{a}}_{x_{k-1}}{\hat{q}}_{3_{k-1}}+{\hat{a}}_{y_{k-1}}{\hat{q}}_{0_{k-1}}+{\hat{a}}_{z_{k-1}}{\hat{q}}_{1_{k-1}}\right)$$

(27) $$A_{58}=2T_{s}\left({\hat{a}}_{x_{k-1}}{\hat{q}}_{2_{k-1}}-{\hat{a}}_{y_{k-1}}{\hat{q}}_{1_{k-1}}+{\hat{a}}_{z_{k-1}}{\hat{q}}_{0_{k-1}}\right)$$

(28) $$A_{59}=2T_{s}\left({\hat{a}}_{x_{k-1}}{\hat{q}}_{0_{k-1}}+{\hat{a}}_{y_{k-1}}{\hat{q}}_{2_{k-1}}+{\hat{a}}_{z_{k-1}}{\hat{q}}_{3_{k-1}}\right)$$

(29) $$A_{5a}=2T_{s}\left({\hat{a}}_{x_{k-1}}{\hat{q}}_{1_{k-1}}-{\hat{a}}_{y_{k-1}}{\hat{q}}_{3_{k-1}}+{\hat{a}}_{z_{k-1}}{\hat{q}}_{2_{k-1}}\right)$$

(30) $$A_{67}=2T_{s}\left({\hat{a}}_{x_{k-1}}{\hat{q}}_{2_{k-1}}-{\hat{a}}_{y_{k-1}}{\hat{q}}_{1_{k-1}}+{\hat{a}}_{z_{k-1}}{\hat{q}}_{0_{k-1}}\right)$$

(31) $$A_{68}=2T_{s}\left({\hat{a}}_{x_{k-1}}{\hat{q}}_{3_{k-1}}-{\hat{a}}_{y_{k-1}}{\hat{q}}_{0_{k-1}}-{\hat{a}}_{z_{k-1}}{\hat{q}}_{1_{k-1}}\right)$$

(32) $$A_{69}=2T_{s}\left({\hat{a}}_{x_{k-1}}{\hat{q}}_{0_{k-1}}+{\hat{a}}_{y_{k-1}}{\hat{q}}_{3_{k-1}}-{\hat{a}}_{z_{k-1}}{\hat{q}}_{2_{k-1}}\right)$$

(33) $$A_{6a}=2T_{s}\left({\hat{a}}_{x_{k-1}}{\hat{q}}_{1_{k-1}}+{\hat{a}}_{y_{k-1}}{\hat{q}}_{2_{k-1}}+{\hat{a}}_{z_{k-1}}{\hat{q}}_{3_{k-1}}\right)$$

(34) $$A_{78}=-\frac{1}{2}T_{s}{\hat{p}}_{k-1}$$

(35) $$A_{79}=-\frac{1}{2}T_{s}{\hat{q}}_{k-1}$$

(36) $$A_{7a}=-\frac{1}{2}T_{s}{\hat{r}}_{k-1}$$

(37) $$A_{87}=\frac{1}{2}T_{s}{\hat{p}}_{k-1}$$

(38) $$A_{89}=\frac{1}{2}T_{s}{\hat{r}}_{k-1}$$

(39) $$A_{8a}=-\frac{1}{2}T_{s}{\hat{q}}_{k-1}$$

(40) $$A_{97}=\frac{1}{2}T_{s}{\hat{q}}_{k-1}$$

(41) $$A_{98}=-\frac{1}{2}T_{s}{\hat{r}}_{k-1}$$

(42) $$A_{9a}=\frac{1}{2}T_{s}{\hat{p}}_{k-1}$$

(43) $$A_{a7}=\frac{1}{2}T_{s}{\hat{r}}_{k-1}$$

(44) $$A_{a8}=\frac{1}{2}T_{s}{\hat{q}}_{k-1}$$

(45) $$A_{a9}=-\frac{1}{2}T_{s}{\hat{p}}_{k-1}$$

Note that $T_{s}$ denotes the discrete sampling time.

Within Equation (13), the nonlinear function *f* is discretized as shown in Equation (46) for the utilization of EKF.

(46) $${\hat{x}}_{k}^{-}=\left[\begin{array}{c} x_{k-1}+V_{x_{k-1}}T_{s}\\ y_{k-1}+V_{y_{k-1}}T_{s}\\ z_{k-1}+V_{z_{k-1}}T_{s}\\ V_{x_{k-1}}^{*}\\ V_{y_{k-1}}^{*}\\ V_{z_{k-1}}^{*}\\ q_{0_{k-1}}+T_{s}\left(-\frac{1}{2}p_{k-1}q_{1_{k-1}}-\frac{1}{2}q_{k-1}q_{2_{k-1}}-\frac{1}{2}r_{k-1}q_{3_{k-1}}\right)\\ q_{1_{k-1}}+T_{s}\left(\frac{1}{2}p_{k-1}q_{0_{k-1}}-\frac{1}{2}q_{k-1}q_{3_{k-1}}+\frac{1}{2}r_{k-1}q_{2_{k-1}}\right)\\ q_{2_{k-1}}+T_{s}\left(\frac{1}{2}p_{k-1}q_{3_{k-1}}+\frac{1}{2}q_{k-1}q_{0_{k-1}}-\frac{1}{2}r_{k-1}q_{1_{k-1}}\right)\\ q_{3_{k-1}}+T_{s}\left(-\frac{1}{2}p_{k-1}q_{2_{k-1}}+\frac{1}{2}q_{k-1}q_{1_{k-1}}+\frac{1}{2}r_{k-1}q_{0_{k-1}}\right) \end{array}\right]+w_{k-1}$$

where

(47) $$V_{x_{k-1}}^{*}=\begin{array}{c} V_{x_{k-1}}+T_{s}a_{x_{k-1}}\left(q_{0_{k-1}}^{2}+q_{1_{k-1}}^{2}-q_{2_{k-1}}^{2}-q_{3_{k-1}}^{2}\right)\\ +2T_{s}a_{y_{k-1}}\left(q_{1_{k-1}}q_{2_{k-1}}-q_{0_{k-1}}q_{3_{k-1}}\right)+2T_{s}a_{z_{k-1}}\left(q_{1_{k-1}}q_{3_{k-1}}+q_{0_{k-1}}q_{2_{k-1}}\right) \end{array}$$

(48) $$V_{y_{k-1}}^{*}=\begin{array}{c} V_{y_{k-1}}+2T_{s}a_{x_{k-1}}\left(q_{1_{k-1}}q_{2_{k-1}}+q_{0_{k-1}}q_{3_{k-1}}\right)\\ +T_{s}a_{y_{k-1}}\left(q_{0_{k-1}}^{2}-q_{1_{k-1}}^{2}+q_{2_{k-1}}^{2}-q_{3_{k-1}}^{2}\right)+2T_{s}a_{z_{k-1}}\left(q_{2_{k-1}}q_{3_{k-1}}-q_{0_{k-1}}q_{1_{k-1}}\right) \end{array}$$

(49) $$V_{z_{k-1}}^{*}=\begin{array}{c} V_{z_{k-1}}+2T_{s}a_{x_{k-1}}\left(q_{1_{k-1}}q_{3_{k-1}}-q_{0_{k-1}}q_{2_{k-1}}\right)\\ +2T_{s}a_{y_{k-1}}\left(q_{2_{k-1}}q_{3_{k-1}}+q_{0_{k-1}}q_{1_{k-1}}\right)+T_{s}a_{z_{k-1}}\left(q_{0_{k-1}}^{2}-q_{1_{k-1}}^{2}-q_{2_{k-1}}^{2}+q_{3_{k-1}}^{2}\right)\\ -T_{s}g \end{array}$$

**Figure 3:**
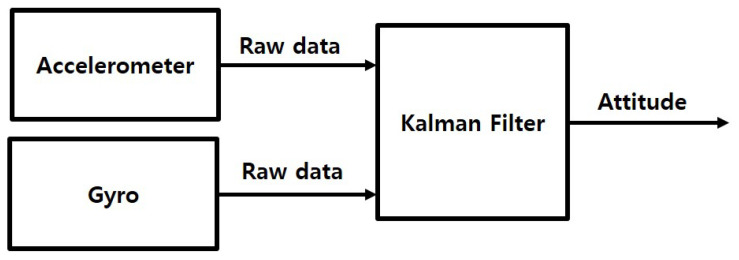
The block diagram of the sensor fusion for attitude estimation.

The IMU of the wearable EPTS considered in this study consists of an accelerometer and a gyroscope. The accelerometer is weak for noise and the gyroscope has a disadvantage of drift error. Therefore, a Kalman filter is used to estimate the attitude of an athlete using the accelerometer and gyroscope data in this study. Figure 3 shows the block diagram of the attitude estimation using an accelerometer and a gyroscope.

The Kalman filter for attitude estimation using an accelerometer and a gyroscope can be represented as:

Prediction equations

(50) $${\hat{x}}_{att_{k}}^{-}=f\left({\hat{x}}_{att_{k-1}},u_{att_{k-1}},0\right)$$

(51) $$P_{att_{k}}^{-}=A_{att_{k}}P_{att_{k-1}}A_{att_{k}}^{T}+Q_{att}$$

Update equations

(52) $$K_{att_{k}}=P_{att_{k}}^{-}H_{att_{k}}^{T}{\left(H_{att_{k}}P_{att_{k}}^{-}H_{att_{k}}^{T}+R_{att}\right)}^{-1}$$

(53) $${\hat{x}}_{att_{k}}={\hat{x}}_{att_{k-1}}^{-}+K_{att_{k}}\left(z_{att_{k}}-h\left({\hat{x}}_{att_{k}}^{-},0\right)\right)$$

(54) $$P_{att_{k}}=\left(1-K_{att_{k}}H_{att_{k}}\right)P_{att_{k}}^{-}$$

where att represents the attitude estimation. The matrix, $A_{att_{k}}$, can be represented as

(55) $$A_{att_{k}}=\left[\begin{array}{cccc} 1 & A_{78} & A_{79} & A_{7a}\\ A_{87} & 1 & A_{79} & A_{8a}\\ A_{97} & A_{98} & 1 & A_{9a}\\ A_{a7} & A_{a8} & A_{a9} & 1 \end{array}\right]$$

## 3. Hardware Experiments

Hardware experiments were performed to validate the performance of the designed GNSS/IMU loosely-coupled integration filter for a wearable EPTS for football players. Rover field tests were first performed, followed by athlete field tests-experiments with an athlete wearing an EPTS. The performance of the wearable EPTS applied to the designed integration filter was compared with that of the performance of the commercial high-precision GNSS/IMU AHRS (Attitude Heading Reference System). Performance analysis of the designed integration filter compared with Vicon data is also provided in this section.

### 3.1. Rover Field Tests

**Figure 4:**
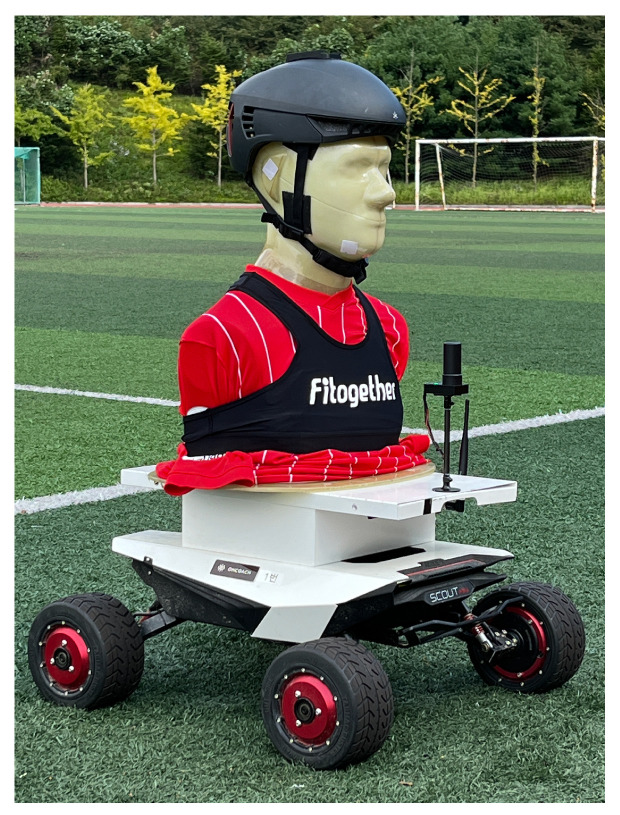
The rover equipped with a wearable EPTS.

**Figure 5:**
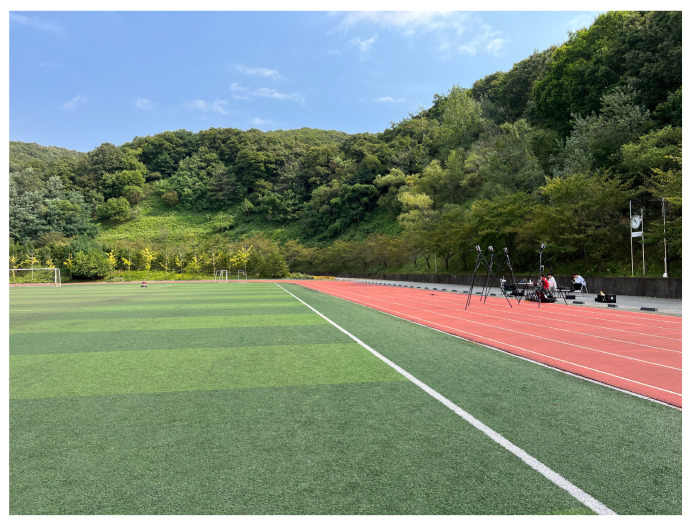
The rover field test at the campus stadium.

An autonomous rover was first considered to validate the performance of the designed GNSS/IMU loosely-coupled integration filter. The considered autonomous rover can control the driving speed, so it is easy to demonstrate the navigation data estimation performance of the designed integration filter. In rover field tests, we focus on the performance of the position and velocity estimation. The rover’s attitude does not change dramatically, and the validation of the attitude estimation performance is not relevant in rover field tests. Figure 4 and Figure 5 show the rover equipped with a wearable EPTS and the test field for rover experiments, respectively.

**Figure 6:**
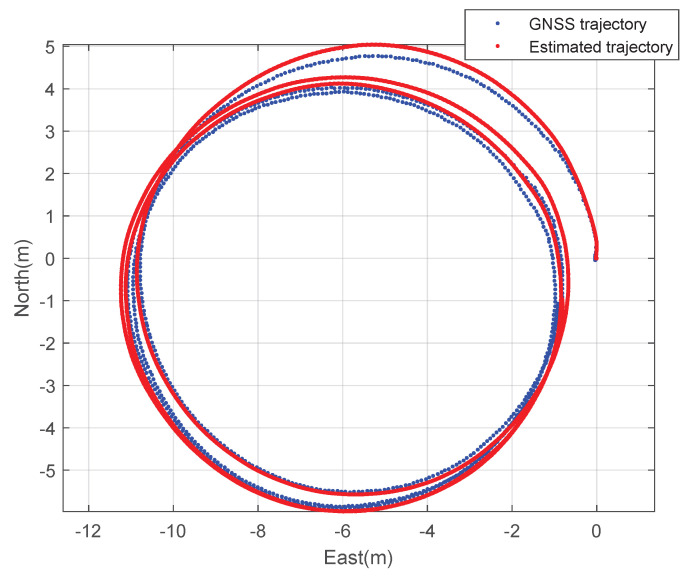
The 2D trajectory of the rover: a circular driving test.

**Figure 7:**
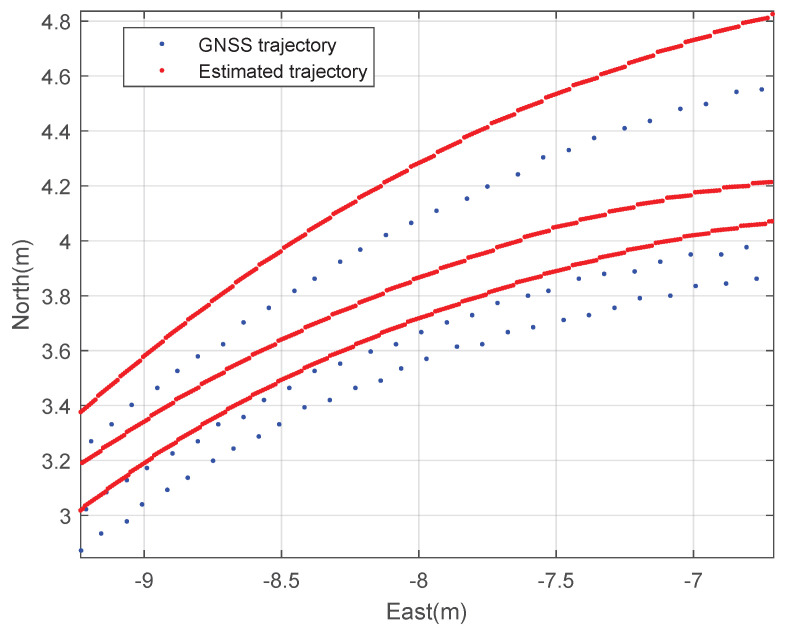
The expanded 2D trajectory of the rover: a circular driving test.

First, constant speed circular driving tests with a 5 m radius were performed. The rover’s speed is 1 m·s^−1^. Figure 6 and Figure 7 show the planar trajectory of the circular driving rover and the expansion of the trajectory of the circular driving rover, respectively. The blue dots represent the measured GNSS trajectory and the red dots represent the trajectory estimated using the designed integration filter. The integration result is much smoother than the GNSS trajectory because the integration data rate is 100 Hz and the GNSS data rate is 10 Hz. This result implies that the designed GNSS/IMU loosely-coupled integration filter can provide instantaneous position, speed, and attitude data. The interpolation of GNSS data can also provide smooth trajectories of athletes. However, obtaining valid instantaneous performance data using the interpolation technique is difficult when athletes move quickly during match games or training sessions.

**Figure 8:**
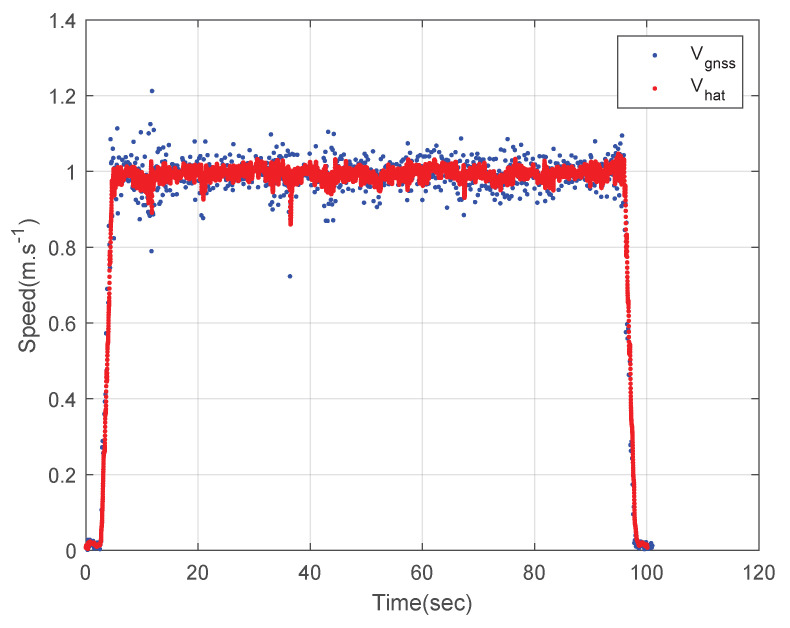
The speed of the rover: a circular driving test.

Figure 8 shows the speed of the circular driving rover. The blue dots represent the measured GNSS speed data and the red dots represent the estimated speed using the designed integration filter. The speed of the rover is estimated as 1 m·s^−1^ using the designed integration filter and the deviation of the integration result is much smaller than that of the GNSS speed data as shown in Figure 8.

**Figure 9:**
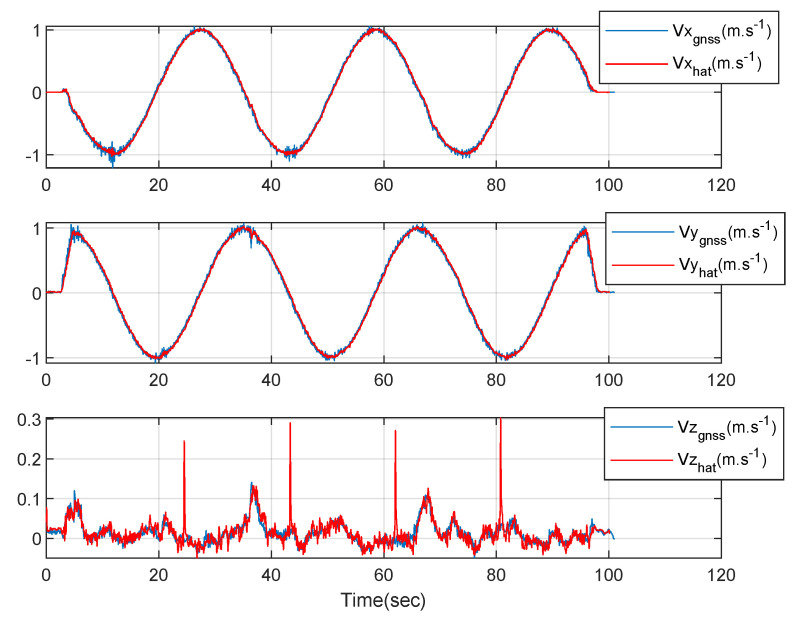
The 3-axis velocities of the rover: a circular driving test.

Figure 9 shows the three-axis velocities of the circular driving rover. The blue lines represent the measured GNSS velocities data and the red lines represent the three-axis velocities estimated using the designed integration filter. The estimated results and the GNSS measured results are similar as shown in Figure 9. There exist four peak points of the z-axis velocity estimated using the designed integration filter due to the field condition.

**Figure 10:**
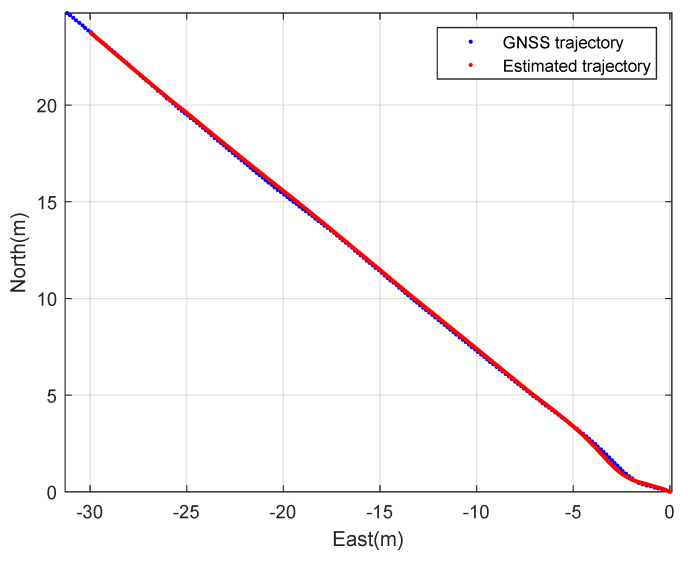
The 2D trajectory of the rover: a straight driving test.

**Figure 11:**
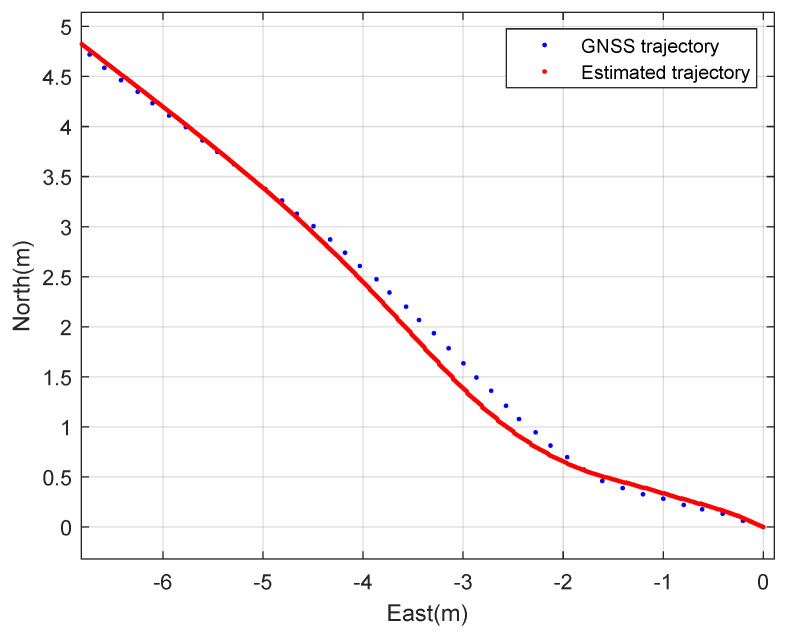
The expanded 2D trajectory of the rover: a straight driving test.

Second, the constant speed straight driving tests were performed. The rover’s speed is 2 m·s^−1^. Figure 10 and Figure 11 show the planar trajectory of the straight driving rover and the expansion of the trajectory of the straight driving rover, respectively. The blue dots represent the measured GNSS trajectory and the red dots represent the trajectory estimated using the designed integration filter. Like the circular driving tests, the integration result is much smoother than the GNSS trajectory.

**Figure 12:**
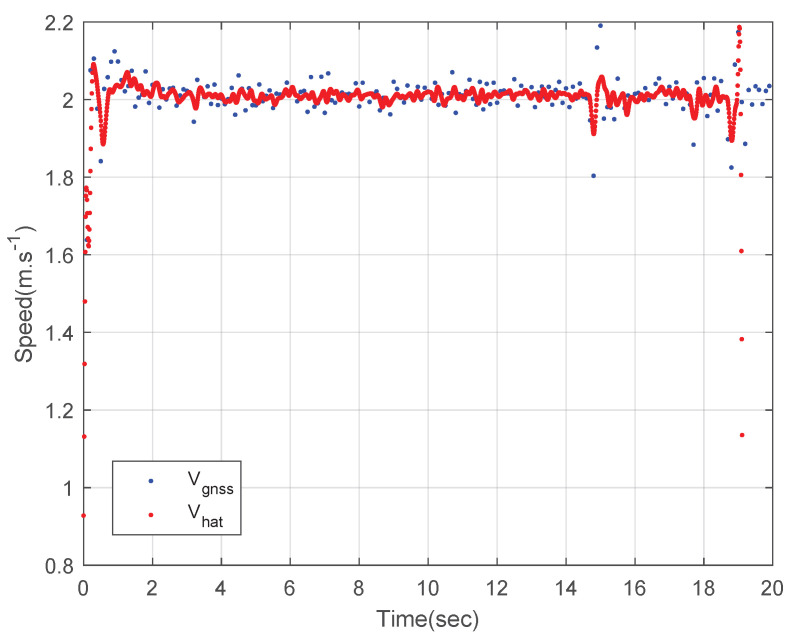
The speed of the rover: a straight driving test.

Figure 12 shows the speed of the straight driving rover. The blue dots represent the measured GNSS speed data and the red dots represent the estimated speed using the designed integration filter. The speed of the rover is estimated as 2 m·s^−1^ using the designed integration filter. Like the circular driving tests, the deviation of the integration result is much smaller than that of the GNSS speed data as shown in Figure 12.

**Figure 13:**
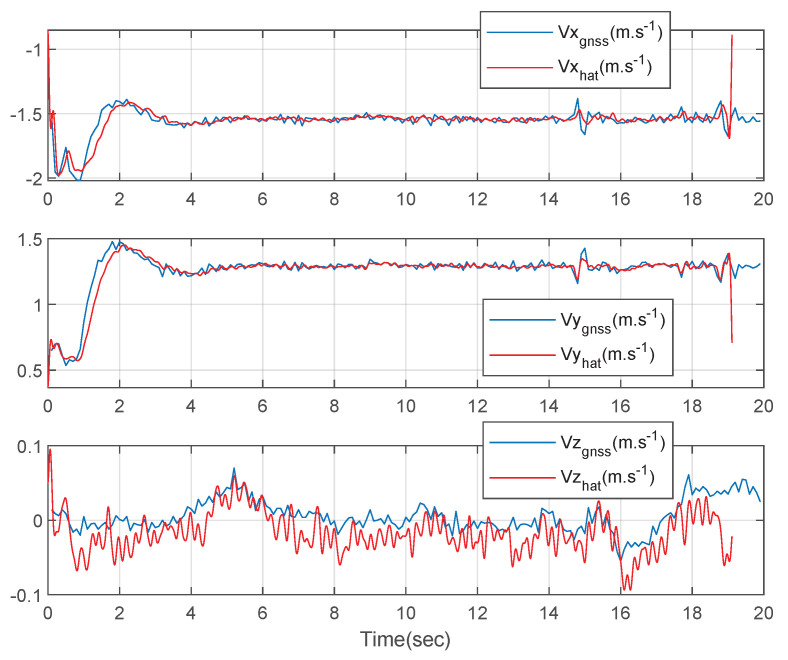
The 3-axis velocities of the rover: a straight driving test.

Figure 13 shows the three-axis velocities of the straight driving rover. The blue lines represent the measured GNSS velocities data and the red lines represent the three-axis velocities estimated using the designed integration filter. The estimated results and the GNSS measured results are similar as shown in Figure 13. There exist four peak points of the z-axis velocity estimated using the designed integration filter due to the field condition.

### 3.2. Athlete Field Tests

**Figure 14:**
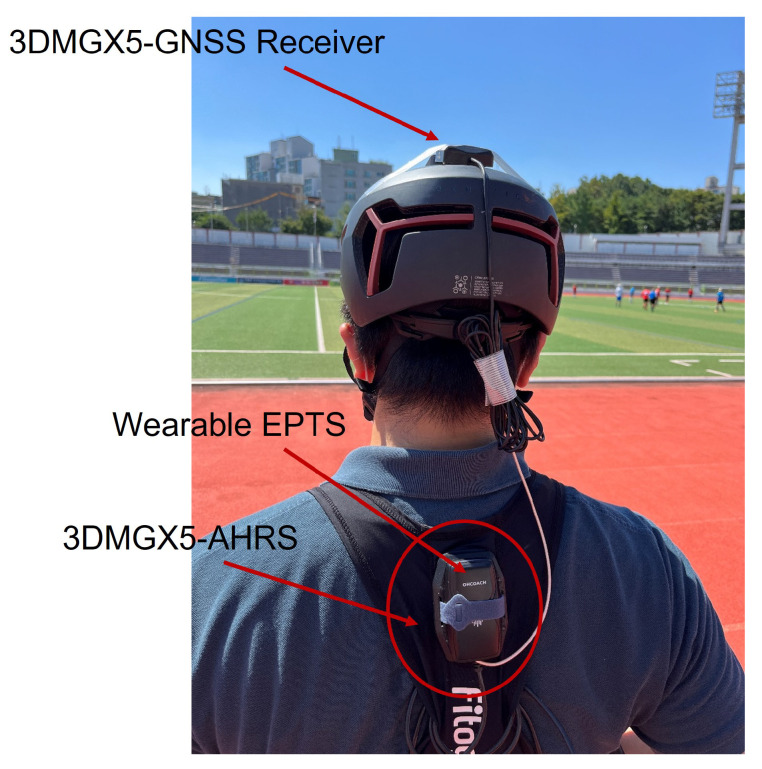
The athlete wearing the wearable EPTS of football player and 3DM-GX5 GNSS/IMU AHRS.

Experiments with an athlete wearing the wearable EPTS were performed to validate the performance of the designed GNSS/IMU loosely-coupled integration filter. A commercial high-precision GNSS/IMU AHRS was used to compare the performance of the designed GNSS/IMU loosely-coupled integration filter. In this study, Microstrain’s 3DM-GX5 GNSS/IMU AHRS is considered a high-precision GNSS/IMU AHRS. Figure 14 shows the athlete wearing the wearable EPTS and Microstrain’s 3DM-GX5 GNSS/IMU AHRS.

**Figure 15:**
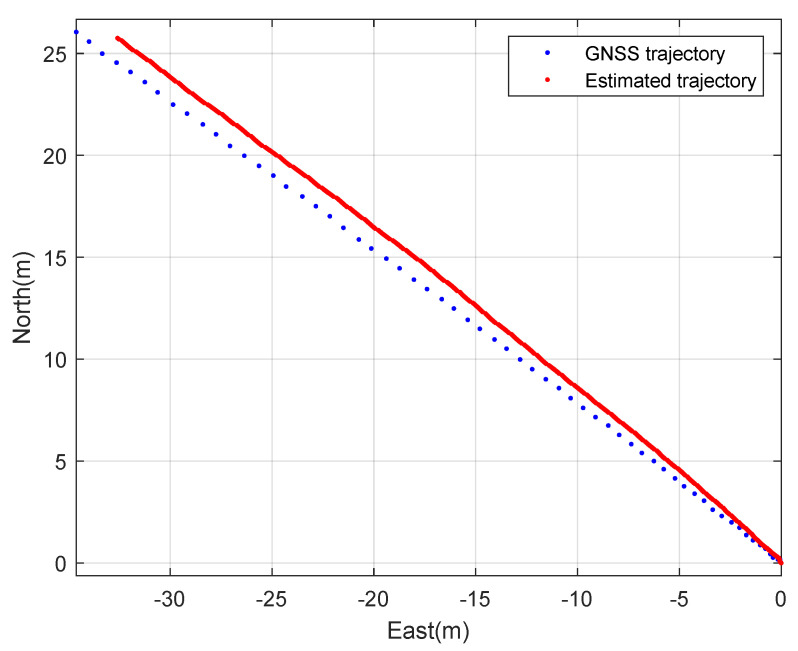
The 2D trajectory of the athlete.

**Figure 16:**
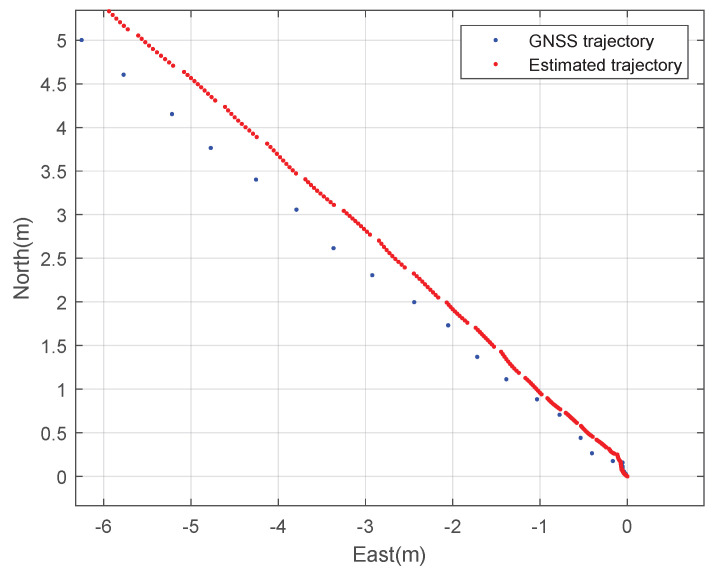
The expanded 2D trajectory of the athlete.

First, the athlete wearing the wearable EPTS spurted straight for 7 s. The athlete’s speed increased to 8 m·s^−1^. Figure 15 and Figure 16 show the planar trajectory of the athlete and the expansion of the trajectory of the athlete, respectively. The blue dots represent the measured GNSS trajectory and the red dots represent the trajectory estimated using the designed integration filter of the wearable EPTS. Like the rover field tests, the integration result is much smoother than the GNSS trajectory, and provides more position data.

**Figure 17:**
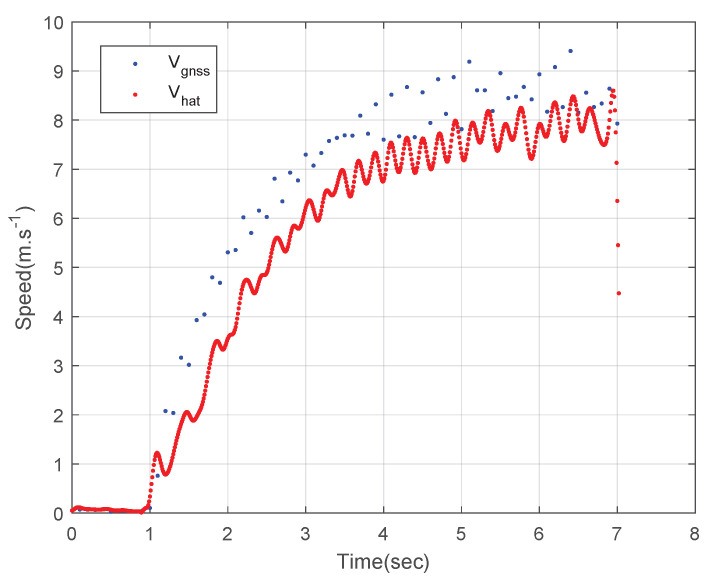
The 2D speed of the athlete.

Figure 17 shows the planar speed of the athlete. The blue dots represent the measured GNSS speed data and the red dots represent the estimated speed of the designed integration filter. Like the rover field tests, the deviation of the integration result is much smaller than that of the GNSS speed data as shown in Figure 17. However, unlike the rover field tests, the speed estimated using the integration filter fluctuated because the athlete’s step affected the inertial sensor measurement when the athlete spurted. This result implies that GNSS/IMU integration can provide the running parameters of athletes, i.e., the number of steps and stride velocity.

**Figure 18:**
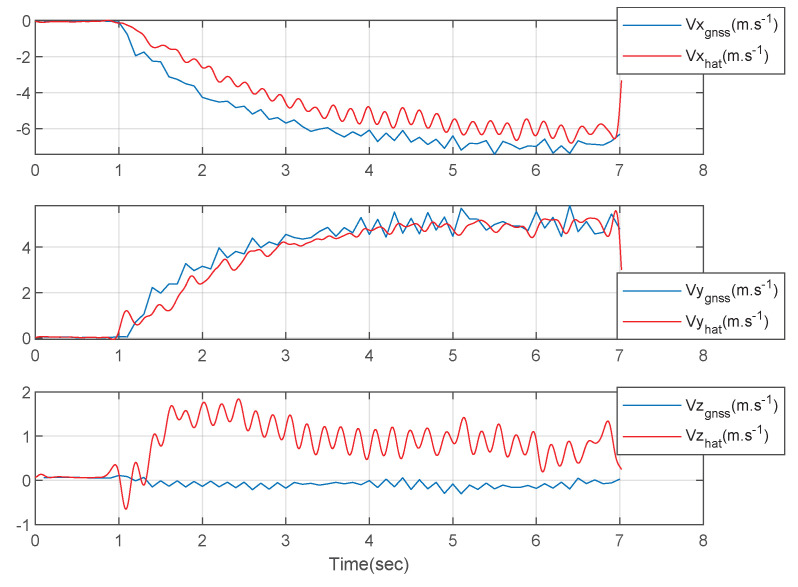
The 3-axis velocities of the athlete.

Figure 18 shows the three-axis velocities of the athlete. The blue lines represent the measured GNSS velocities data and the red lines represent the estimated three-axis velocities using the designed integration filter. The estimated results and the GNSS measured results are similar as shown in Figure 18. The z-axis velocity estimated using the designed integration filter has errors. This is because the IMU in the wearable EPTS might react when the athlete started a spurt.

**Figure 19:**
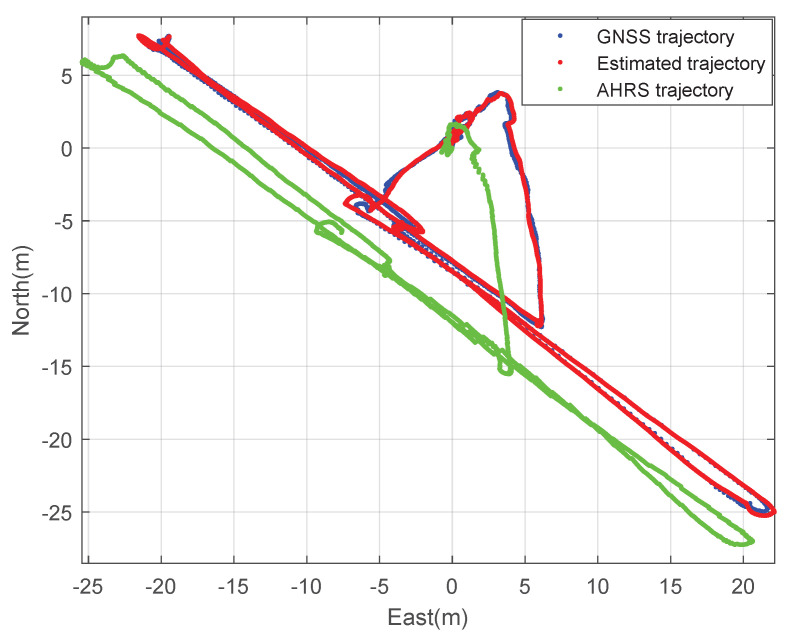
The 2D trajectory of the athlete: comparison with Microstraion’s AHRS.

**Figure 20:**
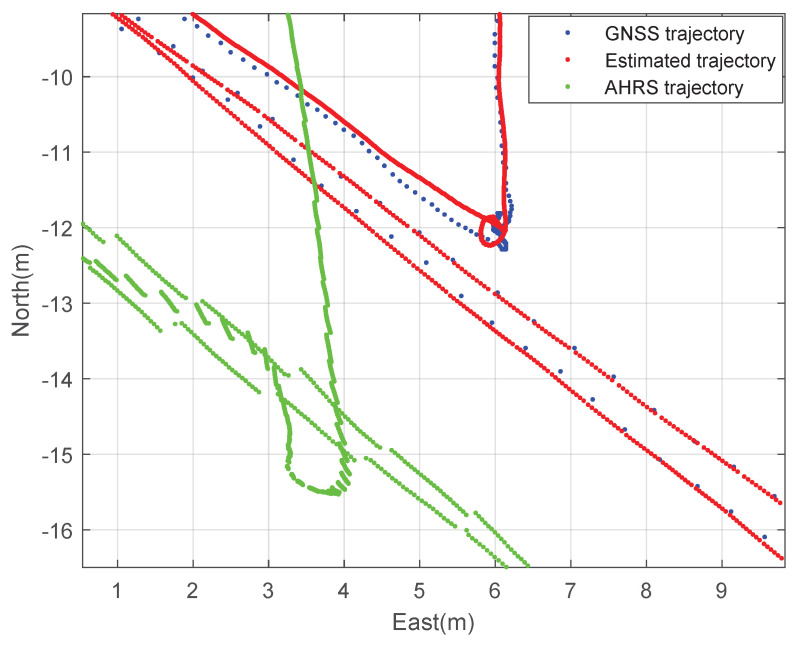
The expanded 2D trajectory of the athlete: comparison with Microstraion’s AHRS.

Second, the performance of the designed GNSS/IMU loosely-coupled integration filter and the commercial high-precision GNSS/IMU AHRS were compared. The athlete ran back and forth at the stadium. Figure 19 and Figure 20 show the planar trajectory of the athlete and the part of the trajectory of the athlete, respectively. The blue dots are the measured trajectory from GNSS in the wearable EPTS, the red dots are the estimated trajectory using the designed integration filter of the wearable EPTS, and the green dots are the estimated trajectory obtained from Microstrain’s 3DM-GX5 GNSS/IMU AHRS. The difference between the estimated position may cause a difference in the GNSS receivers’ specifications. However, overall trajectories are very similar as shown in Figure 19. Moreover, the instantaneous position data estimated using the designed integration filter are much smoother than those of the high-precision GNSS/IMU AHRS as shown in Figure 19.

**Figure 21:**
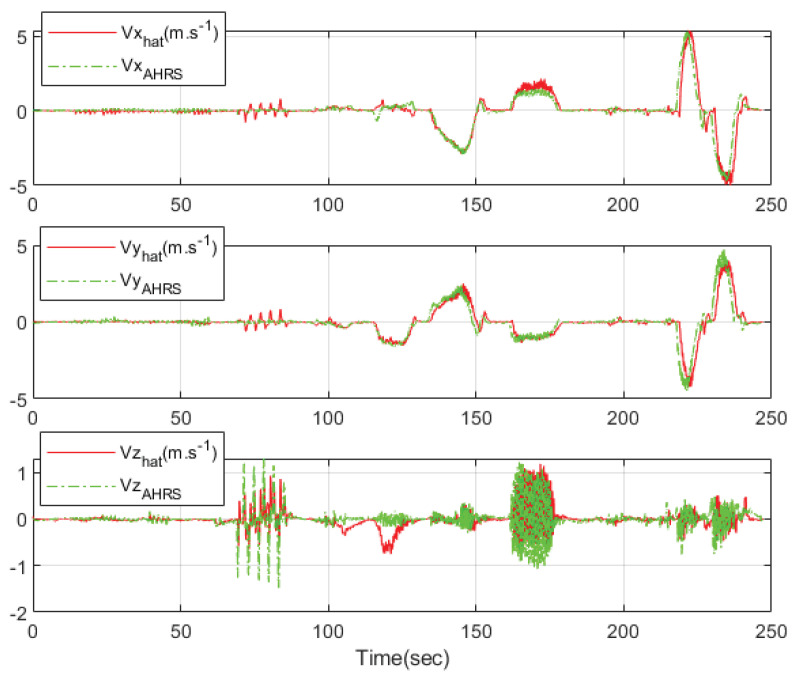
The 3-axis velocities of the athlete: comparison with Microstraion’s AHRS.

Figure 21 shows the three-axis velocities of the athlete for the comparison experiment. The green lines are the estimated three-axis velocities obtained from Microstrain’s 3DM-GX5 GNSS/IMU AHRS and the red lines are the estimated three-axis velocities using the designed integration filter of the wearable EPTS. The estimated results and the high-precision GNSS/IMU AHRS results are similar as shown in Figure 21.

**Figure 22:**
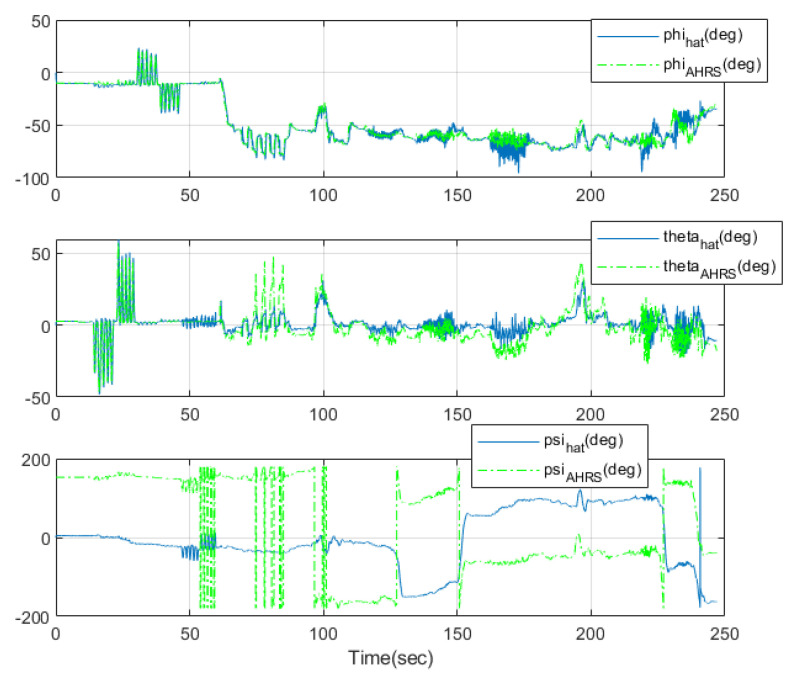
The attitude of the athlete: comparison with Microstraion’s AHRS.

Figure 22 shows the attitude of the athlete for the comparison experiment. The green lines are the estimated attitude of Microstrain’s 3DM-GX5 GNSS/IMU AHRS and the blue lines are the attitude estimated using the designed integration filter of the wearable EPTS. The estimated and high-precision GNSS/IMU AHRS results (except yaw angle) are similar, as shown in Figure 22. The difference in the yaw angle estimates may occur because the gyroscope data of the considered wearable EPTS were only used to estimate the yaw angle. However, the tendencies of the yaw angle estimates are similar. Whether the problem of yaw angle estimation can be solved using magnetometers will be examined in future research.

### 3.3. Vicon Field Test

**Figure 23:**
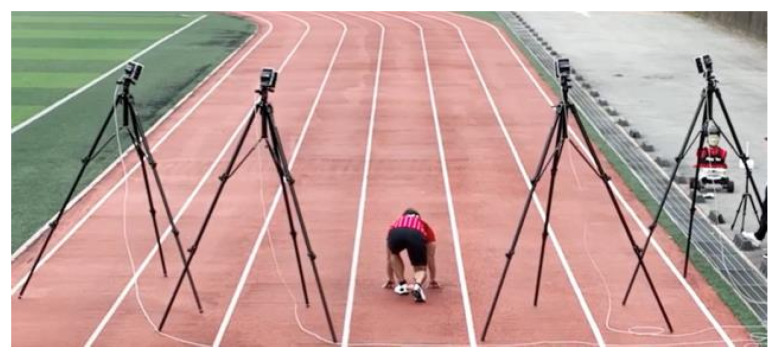
Vicon field tests at the campus stadium.

The experiments with four Vicon motion capture cameras were performed to validate the accuracy of the designed GNSS/IMU loosely-coupled integration filter. The Vicon motion capture camera is a popular, highly accurate system typically used to analyze athlete motion using markers. The Vicon tracking result is considered the ground truth of the player’s motion, with which the accuracy of the designed GNSS/IMU loosely-coupled integration filter was validated. The basic test methods are similar to the previous athlete field tests. The athlete with wearable EPTS sprinted (10 m) six times in a straight line, and the Vicon vantage motion capture camera (located behind the player) recorded the position and three-axis velocities at 100 Hz. Figure 23 shows the athlete wearing OHCOACH Cell, the wearable EPTS, and the campus stadium for Vicon field tests.

**Figure 24:**
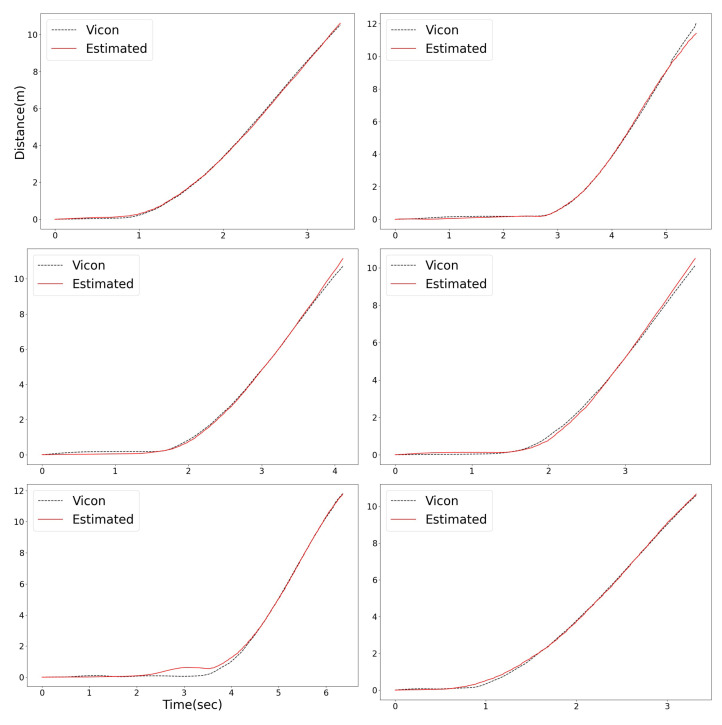
The 2D moving distance of the athlete: comparison with Vicon data.

**Figure 25:**
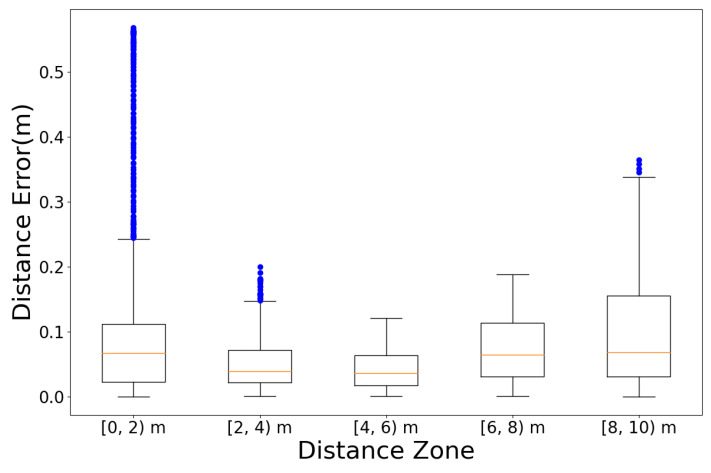
Distance error boxplot by distance zones: comparison with Vicon data.

Figure 24 shows the 2D moving distance of Vicon, estimated using the designed integration filter. The black dashed lines represent the distance captured by Vicon, and the red lines represent the estimated distance from the designed integration filter. We measured the distance error using RMSE (the Root Mean Square Error) metric. The mean, standard deviation, 5% quantile, and 95% quantile distance error depending on the sprinting distance zones are presented in Table 1. In terms of total means, the designed integration filter has distance errors of less than or equal to 10 cm (0.1 m) in every sprinting distance zone, compared with the Vicon ground-truth. This error implies that the distance estimation from the designed integration filter is reliable. From Figure 25, Numerous outliers were observed in the [0, 2) m distance zone, caused by a change in pose from crouch start to running.

**Figure 26:**
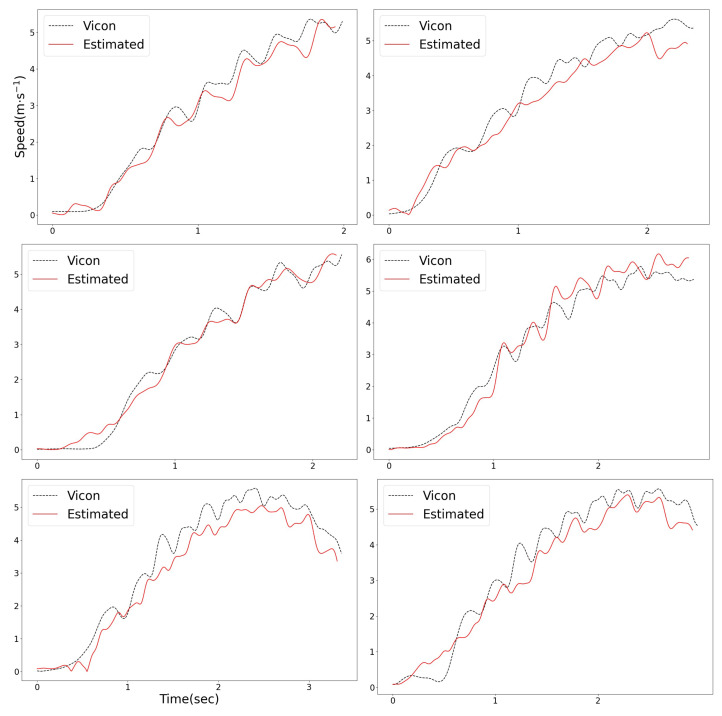
The 2D speed of the athlete: comparison with Vicon data.

**Figure 27:**
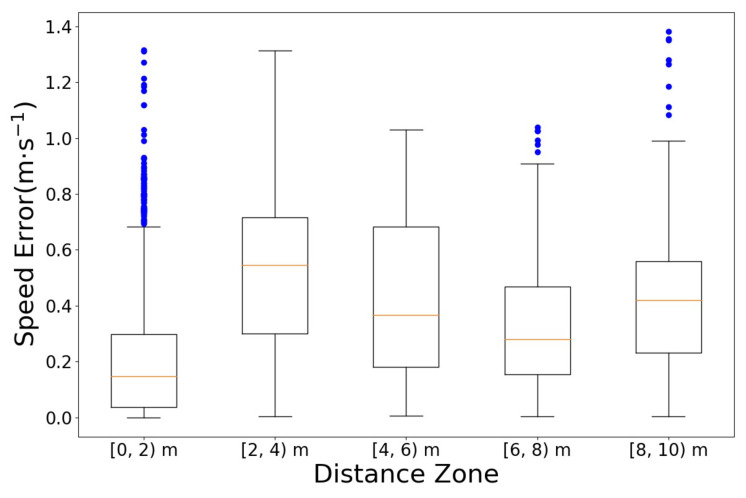
Speed error boxplot by distance zones: comparison with Vicon data.

The speed errors between Vicon and the estimation of the designed integration filter are summarized in Table 2. In terms of total means, the designed integration filter exhibited a speed error of less than or equal to 0.53 m·s^−1^ in every sprinting distance zone, compared with the Vicon ground truth. Figure 26 shows the 2D speed of each system. The black dashed-lines represent the speed captured by Vicon, and the red lines represent the estimated 2D speed of the designed integration filter. For the same reason as the distance error, several outliers occurred in the [0, 2) m distance zone as shown in Figure 27.

## 4. Conclusions

This study presented the GNSS (Global Navigation Satellite System)/IMU (Inertial Measurement Unit) loosely-coupled integration filter for a wearable EPTS (Electronic Performance and Tracking System) for football players. Wearable EPTSs equipped with GNSS and IMU have been widely used to analyze and provide athlete performance data. However, the data obtained from integrating a GNSS and IMU have not yet been used in sports science. Accordingly, an integration filter based on GNSS and IMU data was designed in this study. The loosely-coupled strategy is considered to design the integration filter because of the specifications of the wearable EPTS product. Extended Kalman filters were used to integrate the navigation data of GNSS and the navigation and attitude data of IMU, and quaternion was used to estimate the athlete’s attitude to avoid the gimbal lock singularity in this study. Hardware experiments were performed to validate the performance of the designed GNSS/IMU loosely-coupled integration filter for a wearable EPTS for football players. The comparison results between the designed integration filter for wearable EPTS and a high-precision GNSS/IMU Attitude Heading Reference System and those between the designed integration filter and a Vicon system were also provided to demonstrate the validity of the designed integration filter. Further research is required to improve the performance of the yaw angle estimation using magnetic information, integrate RTK (Real Time Kinetics)-GNSS and IMU, and provide the advanced performance data (e.g., athletes’ steps) obtained from the GNSS/IMU integration.

## Figures and Tables

**Table 1:** Distance error (m) (mean ± standard deviation, [5%, 95% quantiles]): comparison with Vicon data.

Distance Zone	[0, 2) m	[2, 4) m	[4, 6) m	[6, 8) m	[8, 10) m
Sprint 1	0.05 ± 0.02 [0.01, 0.09]	0.03 ± 0.01 [0.01, 0.05]	0.06 ± 0.03 [0.02, 0.10]	0.08 ± 0.02 [0.06, 0.11]	0.05 ± 0.02 [0.01, 0.10]
Sprint 2	0.04 ± 0.04 [0.00, 0.10]	0.02 ± 0.01 [0.00, 0.04]	0.06 ± 0.03 [0.03, 0.12]	0.13 ± 0.03 [0.09, 0.18]	0.08 ± 0.05 [0.09, 0.18]
Sprint 3	0.09 ± 0.04 [0.01, 0.14]	0.07 ± 0.02 [0.04, 0.10]	0.02 ± 0.02 [0.00, 0.06]	0.03 ± 0.03 [0.00, 0.07]	0.15 ± 0.07 [0.07, 0.27]
Sprint 4	0.09 ± 0.05 [0.01, 0.20]	0.13 ± 0.05 [0.04, 0.18]	0.02 ± 0.02 [0.00, 0.07]	0.14 ± 0.03 [0.09, 0.17]	0.27 ± 0.05 [0.18, 0.36]
Sprint 5	0.18 ± 0.18 [0.00, 0.55]	0.04 ± 0.03 [0.01, 0.09]	0.04 ± 0.02 [0.00, 0.08]	0.04 ± 0.02 [0.01, 0.07]	0.01 ± 0.01 [0.00, 0.04]
Sprint 6	0.08 ± 0.06 [0.01, 0.18]	0.04 ± 0.02 [0.00, 0.07]	0.04 ± 0.02 [0.00, 0.08]	0.03 ± 0.02 [0.00, 0.07]	0.06 ± 0.02 [0.02, 0.09]
Total	0.09 ± 0.12 [0.00, 0.39]	0.05 ± 0.05 [0.01, 0.16]	0.04 ± 0.03 [0.00, 0.09]	0.07 ± 0.05 [0.01, 0.16]	0.10 ± 0.10 [0.00, 0.29]

**Table 2:** Speed error (m·s^−1^) (mean ± standard deviation, [5%, 95% quantiles]): comparison with Vicon data.

Distance Zone	[0, 2) m	[2, 4) m	[4, 6) m	[6, 8) m	[8, 10) m
Sprint 1	0.19 ± 0.16 [0.01, 0.48]	0.47 ± 0.33 [0.02, 1.13]	0.37 ± 0.32 [0.07, 1.02]	0.27 ± 0.14 [0.34, 0.56]	0.36 ± 0.17 [0.13, 0.59]
Sprint 2	0.12 ± 0.17 [0.00, 0.52]	0.59 ± 0.15 [0.30, 0.79]	0.36 ± 0.23 [0.04, 0.69]	0.49 ± 0.35 [0.04, 1.03]	0.66 ± 0.09 [0.53, 0.77]
Sprint 3	0.19 ± 0.16 [0.00, 0.51]	0.24 ± 0.16 [0.34, 0.54]	0.27 ± 0.15 [0.03, 0.50]	0.31 ± 0.20 [0.02, 0.80]	0.55 ± 0.46 [0.06, 1.35]
Sprint 4	0.26 ± 0.27 [0.00, 0.86]	0.46 ± 0.27 [0.07, 0.95]	0.29 ± 0.19 [0.01, 0.71]	0.23 ± 0.18 [0.05, 0.56]	0.42 ± 0.16 [0.17, 0.67]
Sprint 5	0.24 ± 0.24 [0.00, 0.78]	0.75 ± 0.30 [0.20, 1.28]	0.69 ± 0.18 [0.36, 0.93]	0.43 ± 0.23 [0.02, 0.74]	0.43 ± 0.08 [0.27, 0.56]
Sprint 6	0.34 ± 0.27 [0.01, 0.74]	0.66 ± 0.26 [0.17, 1.13]	0.56 ± 0.31 [0.03, 0.92]	0.28 ± 0.14 [0.04, 0.51]	0.38 ± 0.21 [0.01, 0.69]
Total	0.21 ± 0.23 [0.00, 0.69]	0.53 ± 0.30 [0.05, 1.07]	0.42 ± 0.28 [0.04, 0.90]	0.34 ± 0.24 [0.04, 0.82]	0.43 ± 0.28 [0.04, 0.91]

## Data Availability

Not applicable.
